# Role of Macrophage CCAAT/Enhancer Binding Protein Delta in the Pathogenesis of Rheumatoid Arthritis in Collagen-Induced Arthritic Mice

**DOI:** 10.1371/journal.pone.0045378

**Published:** 2012-09-24

**Authors:** Ling-Hua Chang, Huei-Sheng Huang, Po-Ting Wu, I-Ming Jou, Min-Hsiung Pan, Wen-Chang Chang, Dennis Ding Hwa Wang, Ju-Ming Wang

**Affiliations:** 1 Institute of Bioinformatics and Biosignal Transduction, National Cheng Kung University, Tainan, Taiwan; 2 Infectious Disease and Signaling Research Center, National Cheng Kung University, Tainan, Taiwan; 3 Institute of Basic Medical Sciences, College of Medicine, Tainan, Taiwan; 4 Department of Medical Laboratory Science and Biotechnology, Tainan, Taiwan; 5 Orthopedics Department of National Cheng Kung University Hospital, Tainan, Taiwan; 6 Graduate Institute of Medical Sciences, College of Medicine, Taipei Medical University, Taipei, Taiwan; 7 Department of Seafood Science, National Kaohsiung Marine University, Kaohsiung, Taiwan; 8 Department of Biological Chemistry, UC Irvine School of Medicine, University of California Irvine, Irvine, California, United States of America; Institute of Environmental Health, United States of America

## Abstract

**Background:**

The up-regulation of CCAAT/enhancer binding protein delta (CEBPD) has frequently been observed in macrophages in age-associated disorders, including rheumatoid arthritis (RA). However, the role of macrophage CEBPD in the pathogenesis of RA is unclear.

**Methodology and Principal Findings:**

We found that the collagen-induced arthritis (CIA) score and the number of affected paws in *Cebpd^−/−^* mice were significantly decreased compared with the wild-type (WT) mice. The histological analysis revealed an attenuated CIA in *Cebpd^−/−^* mice, as shown by reduced pannus formation and greater integrity of joint architecture in affected paws of *Cebpd^−/−^* mice compared with WT mice. In addition, immunohistochemistry analysis revealed decreased pannus proliferation and angiogenesis in *Cebpd^−/−^* mice compared with WT mice. CEBPD activated in macrophages played a functional role in promoting the tube formation of endothelial cells and the migration and proliferation of synoviocytes. *In vivo* DNA binding assays and reporter assays showed that CEBPD up-regulated *CCL20*, *CXCL1*, *IL23A* and *TNFAIP6* transcripts through direct binding to their promoter regions. CCL20, IL23A, CXCL1 and TNFAIP6 contributed to the migration and proliferation of synoviocytes, and the latter two proteins were involved in tube formation of endothelial cells. Finally, two anti-inflammatory chemicals, inotilone and rosmanol, reduced the expression of CEBPD and its downstream targets and mitigated the above phenomena.

**Conclusions and Significance:**

Collectively, our findings suggest that CEBPD and its downstream effectors could be biomarkers for the diagnosis of RA and potentially serve as therapeutic targets for RA therapy.

## Introduction

RA is a chronic autoimmune disease that typically affects the peripheral joints and leads to cartilage and bone destruction [1], [2]. The inflamed synovium consists of fibroblast-like synoviocytes (FLS), which increase greatly in mass and become locally invasive. This invasive and destructive front, called a ‘pannus’, necessitates an increase in the vascular supply to the synovium to cope with the increased requirement for oxygen and nutrients. Angiogenesis is now recognized as a key event in the formation and maintenance of the pannus in RA [3]–[5]. The inflammatory process is characterized by the infiltration of inflammatory cells into the joints. The infiltrating cells, such as macrophages, T cells, B cells, monocytes and dendritic cells, play important roles in the pathogenesis of RA. Activated macrophages produce many pro-inflammatory factors, such as tumor necrosis factor alpha (TNFα) and interleukin-1 beta (IL-1β), which contribute to inflammation and joint destruction [6]. Despite significant advances over the past few years, the pathogenic mechanisms of RA are still obscure, and controversy surrounds the relative contributions of the various cell populations and inflammatory mediators to the disease process [7], [8].

Inflammatory responses originate mainly at the level of transcription [9]. As mentioned above, macrophages play a central role in RA, as they induce the expression of many mediators through paracrine or autocrine effects [10]. The CCAAT/enhancer-binding proteins (C/EBPs) constitute a subfamily of the basic leucine zipper domain transcription factors. Six members have been identified in mammalian cells, including CEBPA, CEBPB, CEBPD, CEBPE, CEBPG and CEBPZ. C/EBPs serve as transcription factors participating in tissue differentiation, metabolism and immune responses. However, their individual roles, especially in macrophages upon inflammation, remain to be explored. CEBPD is expressed at a relatively low level under normal physiological conditions and is up-regulated by a variety of extracellular stimuli, such as interleukin-6 (IL-6), lipopolysaccharide (LPS), IL-1β, interferon-α (IFNα), IFNγ and TNFα [11]–[14]. CEBPD is induced in age-associated disorders, such as Alzheimer’s disease [15], atherosclerosis [16], type 2 diabetes [17] and RA [11]. These discoveries imply that CEBPD may play a central role in inflammatory diseases. However, CEBPD-mediated gene expressions in macrophages are not well understood, nor are their consequent effects in various inflammatory diseases. Therefore, the investigation of macrophage CEBPD-mediated gene expression and cellular functions in the pathogenesis of RA may provide solutions for RA therapy.

## Results

### CEBPD Plays an Important Role in CIA Induction in Mice

**Figure 1:**
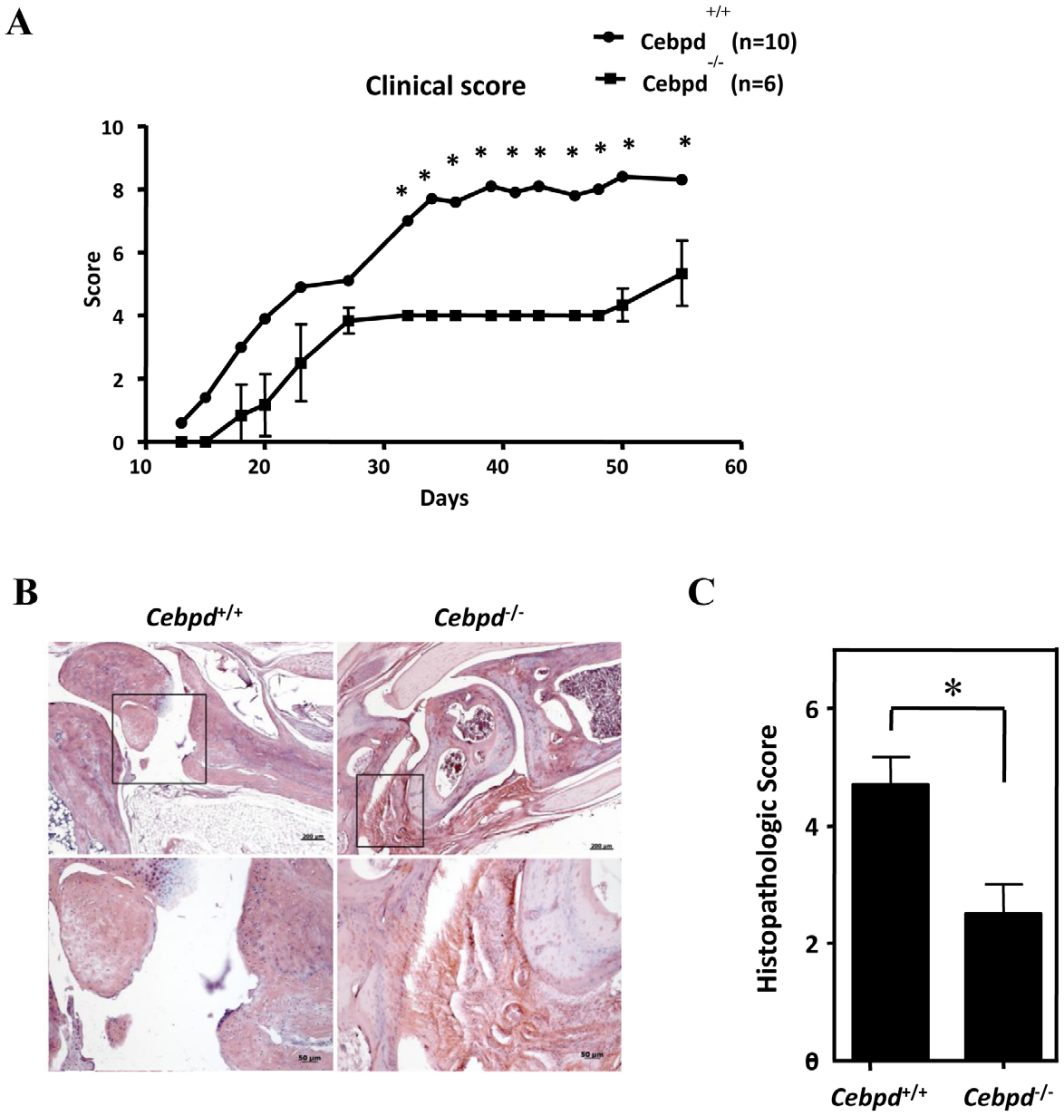
Cebpd plays an important role in CIA mice. A, Age- and sex-matched C57BL/6 mice were immunized with chicken CII emulsified in CFA at day 0. The mice were scored every 2–3 days without knowledge of their genotypes. Clinical score of arthritis: the scores for four paws were obtained for each mouse, and the total severity score for the group was divided by the number of animals in the group to obtain an average severity score. B, The rear paws and joints from *Cebpd^+/+^* and *Cebpd^−/−^* mice were stained with hematoxylin and eosin after collagen-induced arthritis. C, Histopathological scores: the score from two ankle joints in each mouse was assessed by two orthopedic surgeons blind to the genotypes of the mice. The average histopathological score in the *Cebpd ^−/−^* group was significantly lower than that of the wild-type group (*p = *0.016). *p<0.05; *n = *10 for control and *n = *6 for the experimental group.

CIA is an animal model of autoimmunity that has been studied extensively because of the similarities of its symptoms to those of RA, including synovitis, bone erosion and pannus formation. Human CEBPD (CEBPD) and mouse Cebpd (Cebpd) proteins and their gene promoter regions are highly conserved [18], which suggests highly conserved functions and regulation. To assess the role of CEBPD in RA pathogenesis, *Cebpd*-deficient C57BL/6 mice were immunized with type II collagen to induce arthritis. The induction rate and onset time of CIA in WT mice were comparable to those previously reported (60% and 3 to 5 weeks after immunization, respectively [19]). Furthermore, in evaluating the CIA severity using the clinical scores, we found that the symptoms in the *Cebpd*-deficient group were significantly alleviated compared with those of the WT group post-immunization. By the 55th day, the average clinical score of the *Cebpd*-deficient group was 5.3, compared with 8.3 in the WT group (Fig. 1A, *p* = 0.042). Two orthopedic observers (P.T. Wu and I.M. Jou) who were unaware of the genotype of the animals scored the histopathology of the hind limb ankle joint. The severity of the disease as determined by the histological features correlated with the observed visual scores. Pannus formation, destruction of the articular surface and, eventually, ankylosis are hallmarks of RA and were observed in all of the scored arthritic mice. All of the *Cebpd*-deficient mice showed mild to moderate infiltration and synovitis of inflammatory cells with integrity of the joint (Fig. 1B). The mean histopathological score of the *Cebpd*-deficient mice was significantly lower than that of the control group (Fig. 1C, *p = *0.016). The interobserver agreement, evaluated by Spearman correlation analysis, was excellent, with a correlation coefficient (r) of 0.96, *p*<0.001.

### Loss of CEBPD is Coincident with Less Cell Growth and Angiogenesis

**Figure 2:**
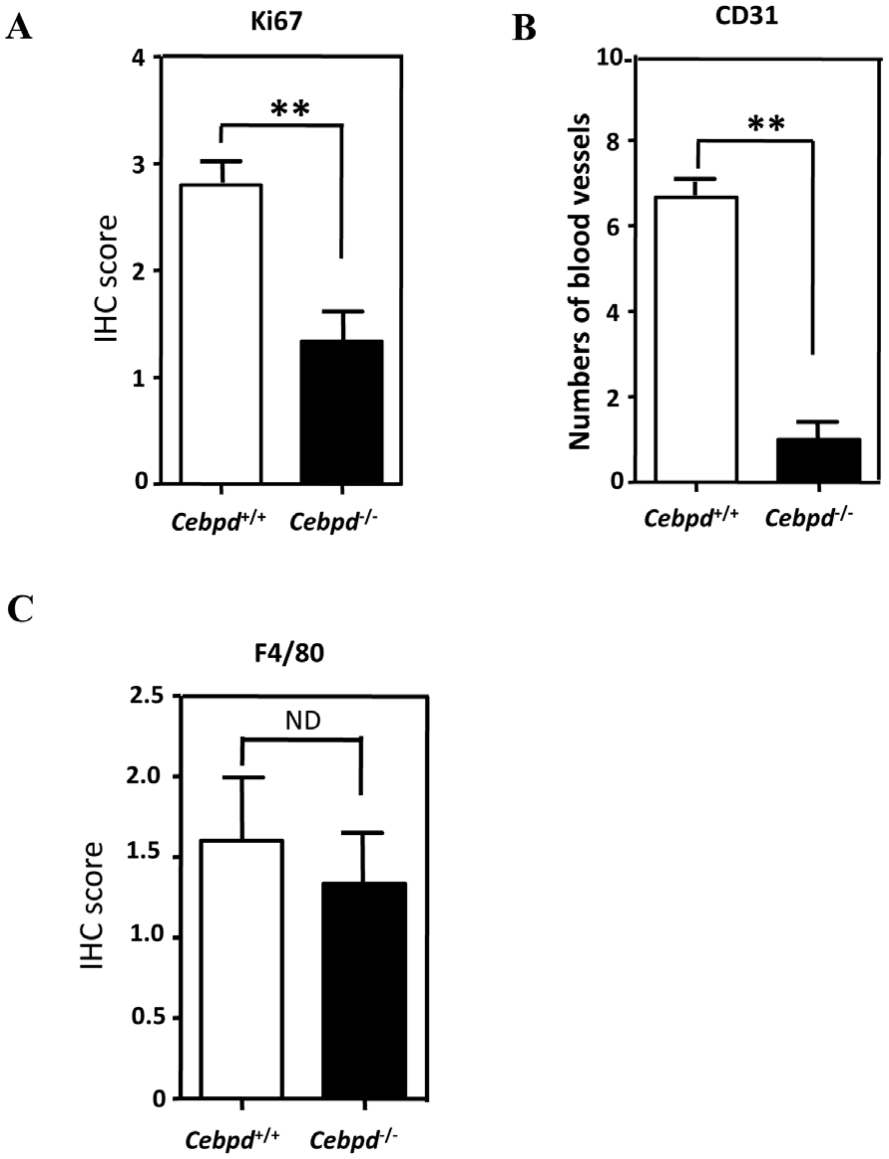
Arthritic pannus and blood vessels are decreased in CIA *Cebpd^−/−^* mice. The rear paws and joints from *Cebpd^+/+^* and *Cebpd^−/−^* mice were immunohistochemically stained after collagen-induced arthritis. A, Ki-67 staining was used as a proliferating cell marker. B, CD31 staining was used as an endothelial cell marker. C, F4/80 staining was used as a macrophage/monocyte marker. ND: no difference. **p<0.01, Student’s t test; *n = *6−10 per group.

Macrophages are principal drivers of synovial inflammation in RA [20]. To assess whether macrophage Cebpd participates in pannus growth, the expression of Ki-67, a proliferation marker, was detected in experimental cells by immunohistochemistry (IHC). Ki-67-positive cells were decreased in *Cebpd*-deficient mice compared with WT mice with CIA (Fig. 2A). Angiogenesis is a key event in the formation and maintenance of the pannus in RA. We therefore assessed whether CEBPD affects angiogenesis by staining for the endothelial cell marker CD31, also named platelet endothelial cell adhesion molecule-1 (PECAM-1). The formation of microvessels showed an 85% reduction in *Cebpd*-deficient mice with CIA (Fig. 2B). This observation suggests that Cebpd participates in the neovascularization of endothelial cells, which may further affect the proliferation of the synovium. Pannus tissue is composed mainly of FLS and macrophages. Examining the accumulation and distribution of macrophages in the pannus following clinical scoring revealed no difference in *Cebpd*-deficient mice and WT mice that were both treated with CIA (Fig. 2C). These data suggest that Cebpd may not affect the recruitment or distribution of macrophages in RA joints.

### Macrophage CEBPD Contributes to the Proliferation and Migration of Synoviocytes

**Figure 3:**
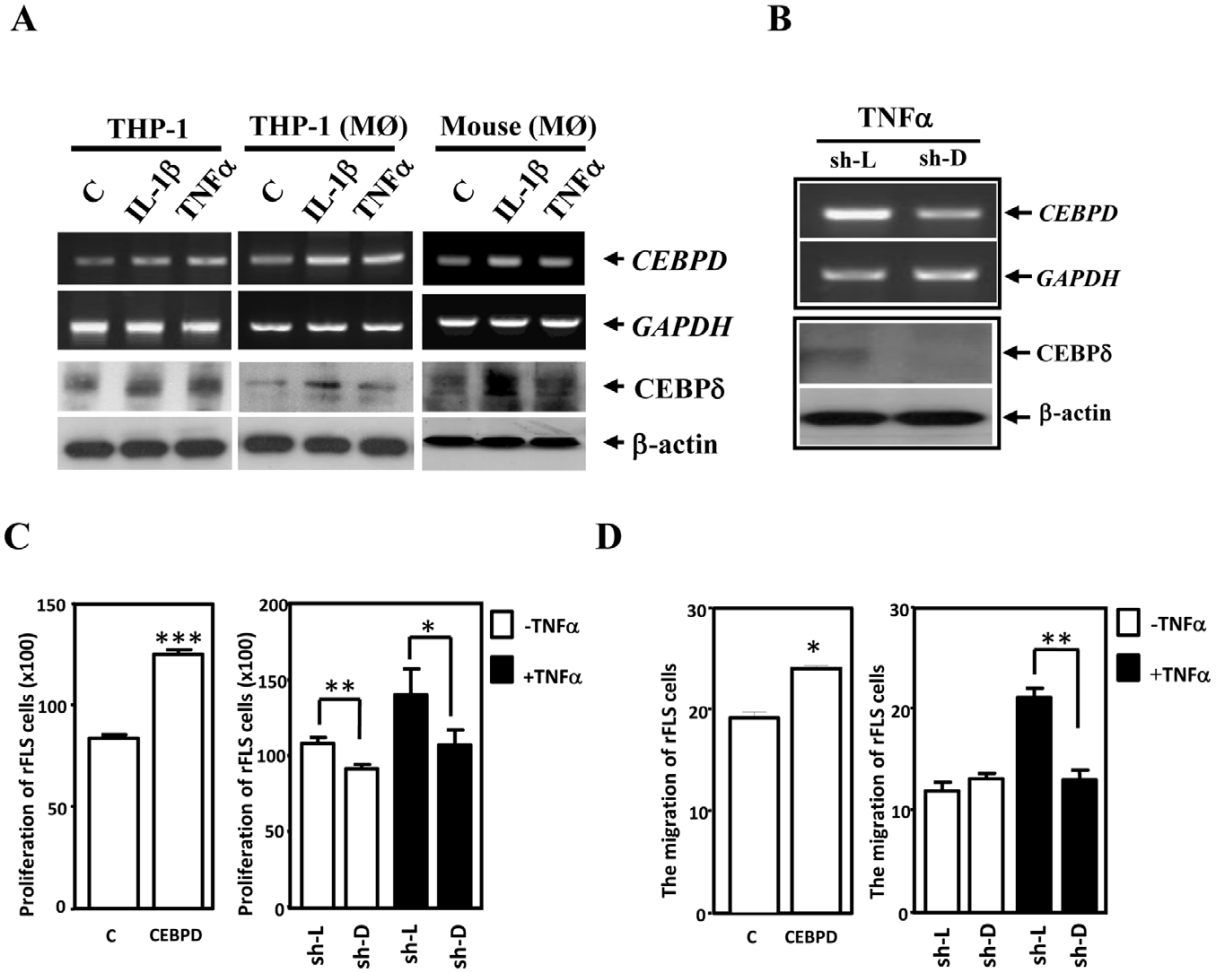
CEBPD promotes rFLS proliferation and migration *in vitro*. A, IL-1β and TNFα induce CEBPD expression in THP-1 cells, THP-1-derived macrophages and mouse primary macrophages. THP-1 cells, THP-1-derived macrophages and mouse primary macrophages were starved in serum-free medium for 6 h and then treated with TNFα or IL-1β for 3 h. Samples of total RNA and cell lysates were harvested for RT-PCR and Western blotting, respectively. B, The effect of lentiviral shCEBPD in THP-1 cells. CEBPD expression upon TNFα treatment and inactivation by lentiviral shCEBPD (sh-D) and shluciferase (sh-L) was examined by RT-PCR and Western blot. *GAPDH* mRNA and β-actin protein were used as the respective internal controls. C-D, Macrophage CEBPD affects the proliferation and migration of rFLS. The conditioned media were harvested as described in the Materials and Methods for further experiments. C, rFLS were treated with conditioned media for 24 h, and the cell viability was measured using a Cell Counting Kit-8. The data are presented as the means ± SE of three independent experiments performed in triplicate. The asterisks represent significant differences (*p<0.05, **p<0.01, ***p<0.001; Student’s t test). D, CM-CEBPD promotes the migration of rFLS cells. The data are presented as the means ± SE of three independent experiments performed in triplicate. The asterisks represent significant differences (*p<0.05, **p<0.01; Student’s t test).

CEBPD was reported to be activated in human RA macrophages [11]. In addition, Cebpd is responsive to LPS treatment in mouse macrophages [14]. However, the response of macrophage CEBPD to the proinflammatory cytokines TNFα and IL-1β is uncertain. The human monocytic leukemia cell line THP-1 is a widely used model for studying monocyte and macrophage biology. Thus, we assessed the responses of *CEBPD* and *Cebpd* transcripts to TNFα and IL-1β treatments in THP-1 cells and primary macrophages from C57BL/6 mice, respectively. As expected, TNFα and IL-1β elevated *CEBPD* and *Cebpd* transcripts and their protein levels in THP-1 cells, phorbol myristate acetate (PMA)-differentiated THP-1 macrophages and mouse primary macrophages (Fig. 3A). This result suggested that the transcriptions of human *CEBPD* and mouse *Cebpd* genes may be similar. The proliferation and migration of FLS are critical signs and symptoms of RA. As shown in Fig. 2, Cebpd contributes to pannus formation and angiogenesis but does not affect macrophage accumulation or distribution in the joints of CIA mice. To assess whether macrophage CEBPD can influence neighboring cells, such as FLS and human umbilical vein endothelial cells (HUVECs), in a paracrine manner, we collected the conditioned medium from CEBPD-overexpressing or CEBPD-knockdown THP-1 cells for further assays. When growing rat FLS (rFLS) in conditioned medium harvested from CEBPD-overexpressing THP-1 cells (CM-CEBPD), we found that CEBPD activation in THP-1 cells promoted the proliferation and migration of rFLS (left panels of Fig. 3C and 3D). In contrast, when growing rFLS in conditioned medium from TNFα-treated THP-1 cells infected with lentivirus expressing shCEBPD (CM-shD) or shLuciferase (CM-shL) (Fig. 3B), we found that the loss of CEBPD attenuated the TNFα-induced proliferation and migration of rFLS (right panels of Fig. 3C and 3D).

### Endothelial Cell Tube Formation Responds to CEBPD-modulated Conditioned Medium

**Figure 4:**
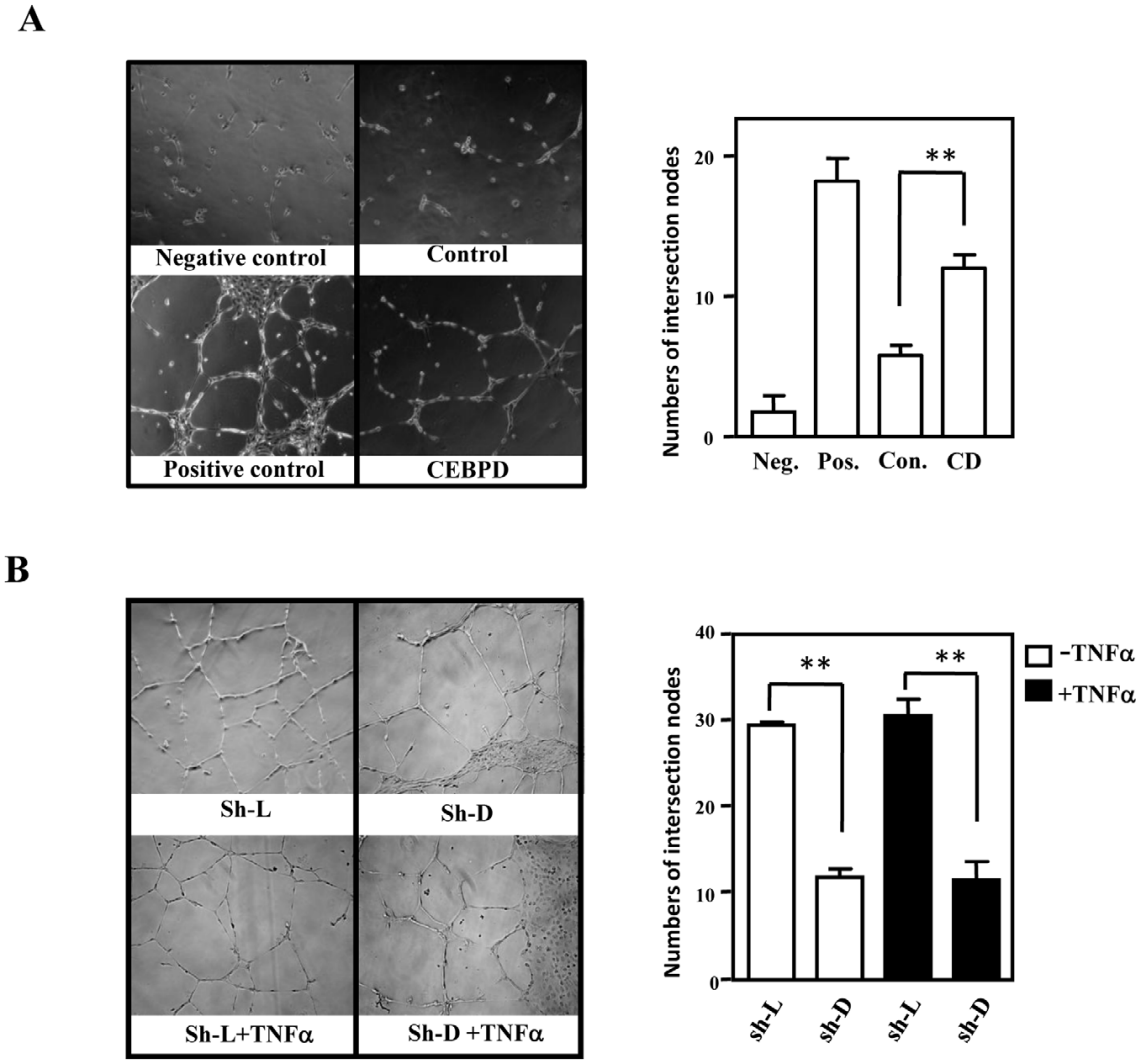
CEBPD promotes endothelial cell tube formation. A, The angiogenic effect of CM-CEBPD on HUVECs. The positive control is HUVECs that were treated with EGM-2. The negative control is HUVECs that were treated with serum-free EBM-2 medium. B, The angiogenic effects of exposing HUVECs for 24 h to conditioned medium from THP-1 cells that were infected with lentivirus shCEBPD (CM-shD) or shLuciferase (CM-shL) with or without TNFα. The number of intersections between branches of assembled endothelial cell networks was counted in the whole field. The data are presented as the means ± SE of three independent experiments performed in triplicate. The asterisks represent significant differences (*p<0.05, **p<0.01; Student’s t test).

Following the same approaches, we tested whether CM-CEBPD affected the proliferation and angiogenesis of HUVECs. The CM-CEBPD promoted the proliferation of HUVECs (left panel of Fig. S1). In contrast, when growing HUVECs in CM-shD or CM-shL from TNFα-treated THP-1 cells, we found that the loss of CEBPD attenuated the TNFα-induced proliferation of HUVECs (right panel of Fig. S1). Moreover, using the tube formation assay, HUVECs were seeded onto Matrigel and grown in CM-CEBPD to assess the angiogenic effect. Measuring the number of intersection formations, the CM-CEBPD showed a pro-angiogenic effect on HUVECs (Fig. 4A). Next, we assessed the effects of CM-shD and CM-shL on the tube formation activity of HUVECs. Compared with CM-shL, CM-shD showed a decrease in endothelial tube formation (Fig. 4B), suggesting that CEBPD activation in macrophages can support angiogenesis.

### Identification of CEBPD Downstream Targets in Macrophages

**Figure 5:**
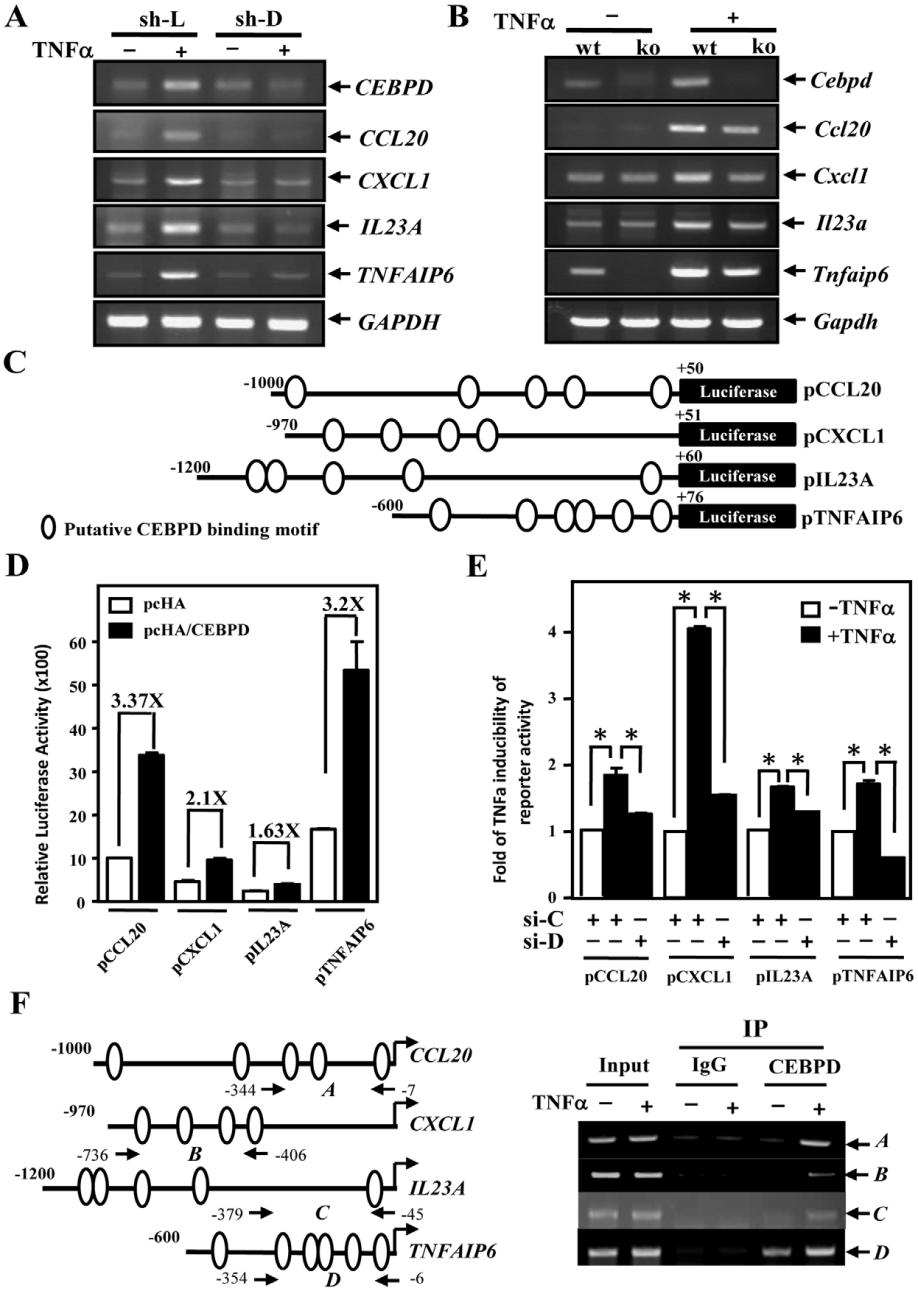
*CCL20*, *CXCL1*, *IL23A* and *TNFAIP6* genes are responsive to CEBPD activation in THP-1 cells. A, CEBPD participates in TNFα-induced *CCL20*, *CXCL1*, *IL23A* and *TNFAIP6* transcripts in THP-1 cells. Total RNA was extracted from TNFα-treated THP-1 cells treated with lentiviral shCEBPD (sh-D) or shluciferase (sh-L) for RT-PCR analysis. B, *Ccl20*, *Cxcl1*, *Il23a* and *Tnfaip6* transcripts in TNFα-treated primary macrophages of WT or *Cebpd*-deficient mice. Total RNA was extracted from TNFα-treated macrophages of WT or *Cebpd*-deficient mice for RT-PCR analysis. C, Putative CEBPD-binding motifs in the *CCL20*, *CXCL1*, *IL23A* and *TNFAIP6* reporters. The 5′-flanking regions of human *CCL20*, *CXCL1*, *IL23A* and *TNFAIP6* genes were subcloned into pGL3 basic reporters. (D) CEBPD activates *CCL20*, *CXCL1*, *IL23A* and *TNFAIP6* reporter activities in THP-1 cells. pcDNA3/HA/CEBPD (pcHA/CEBPD) or pcDNA3/HA (pcHA) was cotransfected with the *CCL20* (−1000 to +50), *CXCL1* (−970 to +51), *IL23A* (−1200 to +60) or *TNFAIP6* (−600 to +76) reporter. The data are presented as the means ± SE of relative luciferase activity from three independent experiments in duplicate. E, CEBPD contributes to TNFα-induced *CCL20*, *CXCL1*, *IL23A* and *TNFAIP6* reporter activities in THP-1 cells. Oligonucleotides of siCEBPD (si-D) or siLacZ (si-C) were cotransfected with various indicated reporters. The lysates of transfectants were harvested after treating with TNFα for 6 h. The data are presented as the means ± SE of relative luciferase activity. The asterisks represent significant differences (*p<0.05; Student’s t test). F, CEBPD binds to the 5′-flanking regions of the *CCL20*, *CXCL1*, *IL23A* and *TNFAIP6* genes *in vivo*. Chromatin was isolated for ChIP analysis from THP-1 cells with or without TNFα treatment, as described in the Materials and Methods. The locations of the PCR primers in the 5′-flanking regions of *CCL20* (−344 to −7), *CXCL1* (−736 to −406), *IL23A* (−379 to −45) and *TNFAIP6* (−354 to −6) are presented in the left panel. Chromatin was separately immunoprecipitated with control IgG and CEBPD antibodies and then amplified by PCR with the indicated primers, as shown in the right panel.

To identify the secretory factors regulated by CEBPD in macrophages, two arrays, a cytokine array and an mRNA microarray, were used. Conditioned medium from THP-1 cells transiently transfected with a CEBPD expression vector was harvested for the cytokine array. The transcripts of THP-1 cells infected with lentiviruses bearing shCEBPD or shLuciferase and then treated with TNFα were collected for the mRNA microarray. In the cytokine array, 25 up-regulated and 24 down-regulated proteins (>2.0-fold, p<0.05) were identified (Table 1). Among these proteins were several important pro-inflammatory factors that have been identified in RA joints, including IFN-γ, IL-1β, IL-6, IL-8, IL-17, IL-23, CCL20, CXCL1, CXCR2 and TGFβ. In addition, over 400 genes (>1.5-fold, p<0.05) were considered to be significant in the mRNA microarray. Comparing the cytokine array and the mRNA microarray, four common genes, *CCL20*, *CXCL1*, *IL23A* and *TNFAIP6*, were identified for further investigation (Table S1). First, we assessed whether these genes are mediated by CEBPD regulation upon TNFα treatment. We found that the transcripts of *CCL20*, *CXCL1*, *IL23A* and *TNFAIP6* were increased by TNFα treatment. However, silencing CEBPD expression using lentiviral shCEBPD attenuated the TNFα-induced *CCL20*, *CXCL1*, *IL23A* and *TNFAIP6* mRNA levels (Fig. 5A). We further assessed these genes in isolated primary macrophages from WT and *Cebpd*-deficient mice. The RT-PCR results show that the TNFα responses of mouse *Ccl20*, *Cxcl1*, *Il23a* and *Tnfaip6* transcripts were attenuated in macrophages of *Cebpd*-deficient mice (Fig. 5B).

**Table 1:** Immune factors responding to the induction of CEBPD in macrophages.

Up		Down	
Names	Folds	Names	Folds
CXCR2/IL-8 RB	2.19	Activin A	0.28
Dtk	2.14	Angiopoietin-1	0.39
Glucagon	2.06	CCR4	0.20
HB-EGF	2.38	DcR3/TNFRSF6	<0.01
IFN-gamma	2.26	GDF5	0.41
GFBP-3(IGFBP-3)	2.08	IL24	0.00
Gro-a (CXCL1)	2.12	Insulin R	0.04
IL-1 beta	3.07	Krmen-1	0.06
IL23A	2.1	LIGHT/TNFSF14	0.01
IL-3	12.99	MIG	0.01
IL-4 R	2.51	MSP alpha	<0.01
IL-5 R a	18.62	NRG2	0.06
IL-6	11.13	Neurturin	<0.01
IL-6 R	74.84	NT-3	<0.01
IL-7	5.8	OSM	0.02
IL-8	4.25	sFRP-1	<0.01
IL-17B R	2.41	Soggy-1	0.02
IL-17D	2.46	TACI/TNFRS13B	0.07
IL17-E	2.91	Thrombopoietin	<0.01
IL-19	2.58	TRADD	0.32
MIP-3 a (CCL20)	2.28	TREM-1	0.22
Siglec-5/CD170	1673	uPAR	<0.01
TGF-beta 3	2.22	Vasorin	<0.01
Pentraxin3/TSG-14	2.67	VEGF R3	0.03
TRAIL R2 DR5/TNFRSF10B	8.88		

Conditioned medium was harvested from THP-1 cells that had been transiently transfected with a CEBPD expression vector for a cytokine array.

Moreover, to assess whether these CEBPD-activated genes are regulated through increased promoter activity, their promoter regions were individually cloned into a reporter vector. Additionally, the putative CEBPD-binding motifs were predicted by the Prediction of CEBPD-Binding Motifs (PCDBM): http://140.116.235.104/PCDBM/program [21] and TFsearch software (http://www.cbrc.jp/research/db/TFSEARCH.html) (Fig. 5C). Next, a reporter assay showed that CEBPD can transactivated the *CCL20*, *CXCL1*, *IL23A* and *TNFAIP6* reporters in THP-1 (Fig. 5D) and U937 cells (Fig. S2). Moreover, attenuated TNFα-induced *CCL20*, *CXCL1*, *IL23A* and *TNFAIP6* reporter activities were observed in the cells cotransfected with the siLacZ or siCEBPD oligonucleotides (Fig. 5E). Additionally, an *in vivo* DNA-binding assay was conducted to assess whether CEBPD could directly bind to the *CCL20*, *CXCL1*, *IL23A* and *TNFAIP6* promoters. The chromatin immunoprecipitation (ChIP) results show that TNFα induced CEBPD binding activity in the *CCL20*, *CXCL1*, *IL23A* and *TNFAIP6* promoters (Fig. 5F).

### The Effects of CCL20, CXCL1, IL23A and TNFAIP6 on the Migration and Proliferation of rFLS and the Tube Formation Ability of HUVECs

**Figure 6:**
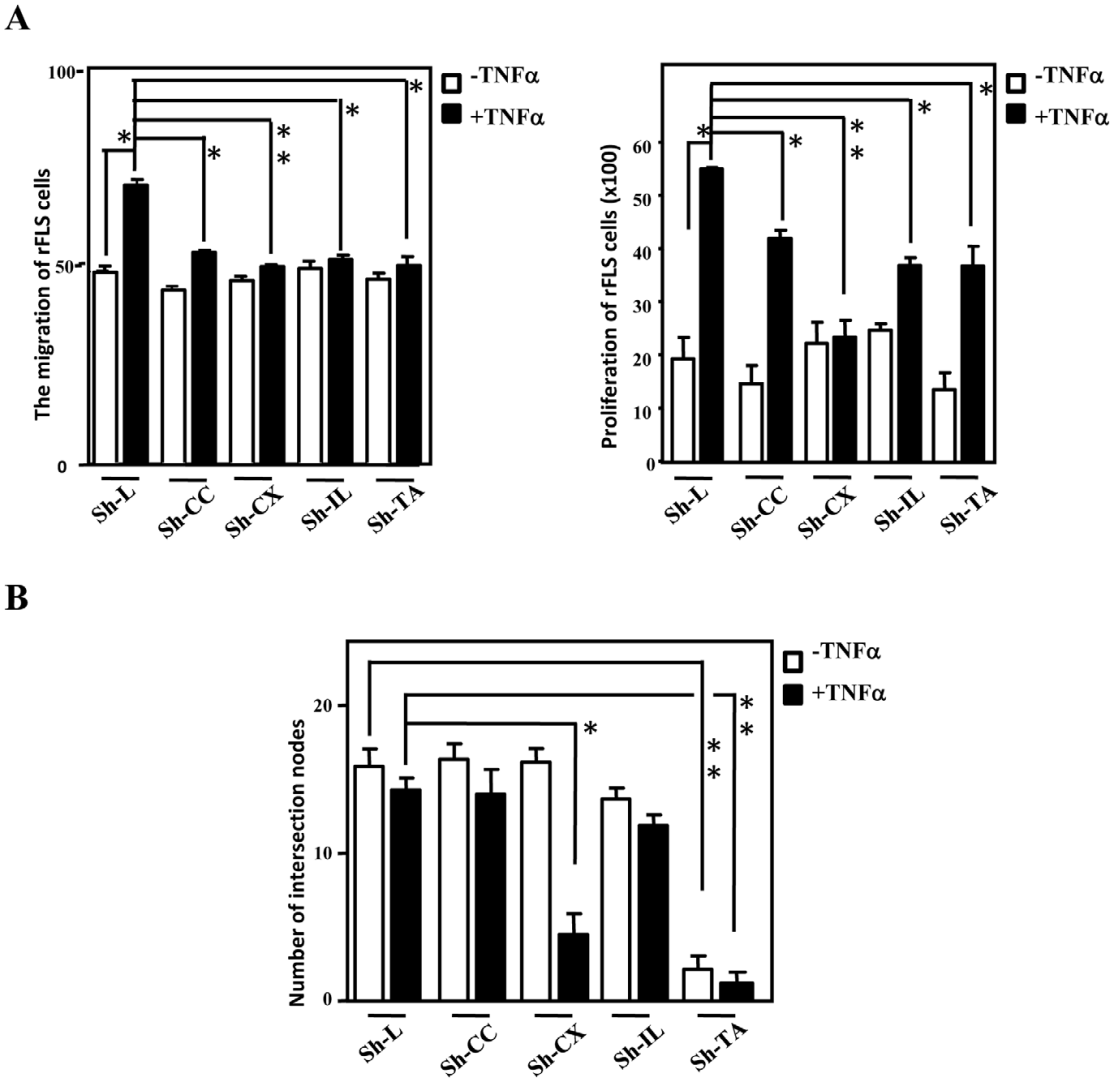
CCL20, CXCL1, IL23A and TNFAIP6 contribute to the proliferation and migration of rFLS cells, but only CXCL1 and TNFAIP6 increase endothelial cell tube formation. A, CCL20, CXCL1, IL23A and TNFAIP6 contribute to the proliferation and migration of rFLS cells. The purified rFLS were cultured with the conditioned medium harvested from TNFα-treated THP-1 cells containing the indicated lentivirus, namely shLuciferase (Sh-L), shCCL20 (Sh-CC), shCXCL1 (Sh-CX), shIL23A (Sh-IL) or shTNFAIP6 (Sh-TA). The migration and proliferation assays were conducted using a Boyden chamber assay and a CCK-8 kit, respectively. The results are shown as the means ± SE. The asterisks represent significant differences (*p<0.05; Student’s t test). B, The endothelial tube formation assay was conducted using conditioned media harvested from THP-1 cells as described in the Materials and Methods. The results are shown in the upper panel and compared statistically in the lower panel. The number of intersections between branches of assembled endothelial cell networks was counted in the whole field. The data are presented as the means ± SE. The asterisks represent significant differences (*p<0.05, **p<0.01; Student’s t test).

Although IL23A plays an important role in the pathogenesis of RA, the functions of the other three CEBPD-responsive candidates remain unclear. A gain-of-function approach with lentiviral shRNAs against CCL20, CXCL1, IL23A and TNFAIP6 was applied in THP-1 cells. After confirming the knockdown efficiency in THP-1 macrophages treated with TNFα or not (Fig. S2), the conditioned media were harvested for further assays. Using the same approaches as shown in Fig. 3, we found that all four proteins contributed to the migration (Fig. 6A, left panel) and proliferation of rFLS (Fig. 6A, right panel). Interestingly, the endothelial tube formation assay showed that the loss of CXCL1 and TNFAIP6, but not CCL20 or IL23A, attenuated the tube formation effect upon TNFα treatment (Fig. 6B).

### The Anti-inflammatory Chemicals Inotilone and Rosmanol Inhibit the Proliferation and Migration of rFLS and the Angiogenesis of HUVECs

**Figure 7:**
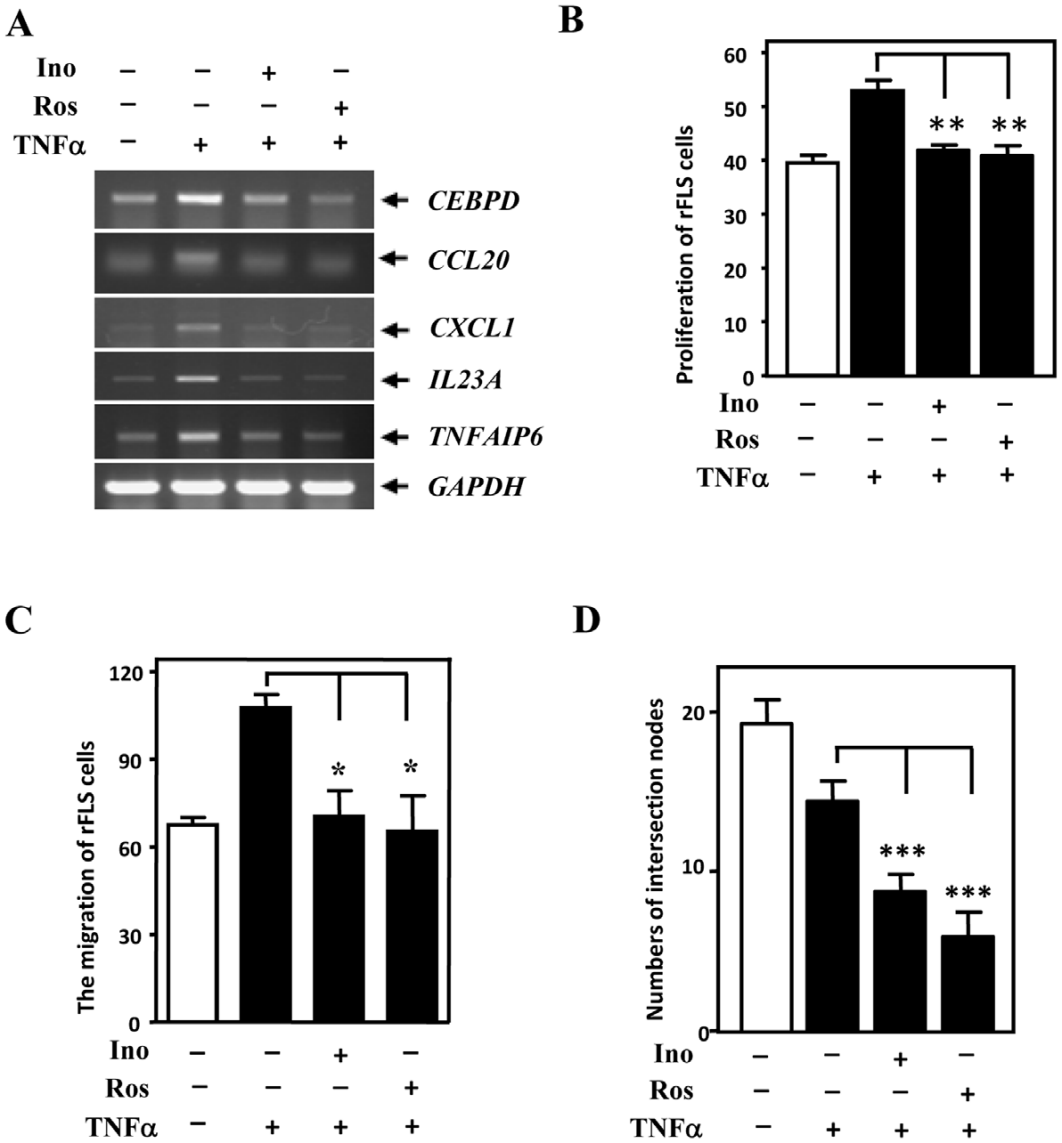
Inotilone and rosmanol inhibit the proliferation and migration of rFLS and the endothelial tube formation of HUVECs. A, Inotilone and rosmanol inhibit *CEBPD*, *CCL20*, *CXCL1*, *IL23A* and *TNFAIP6* transcription in THP-1 cells. Total RNA was harvested from THP-1 cells pretreated with inotilone (Ino) or rosmanol (Ros) then treated with 20 ng/ml TNFαas described in the Materials and Methods. B-D, Inotilone and rosmanol inhibit the proliferation and migration of rFLS and endothelial cell tube formation. The conditioned media were harvested to assess their effects on the proliferation and migration of rFLS and on the endothelial tube formation of HUVECs. The data are presented as the means ± SE of three independent experiments performed in triplicate. The asterisks represent significant differences (*p<0.05, **p<0.01; Student’s t test).

The above results suggested that CEBPD and its downstream targets could be diagnostic markers in RA pathogenesis. Rosmanol is a natural polyphenol derived from the herb rosemary (*Rosmarinus officinalis* L.) that has high antioxidant activity [22]. Inotilone, an unusual 5-methyl-3(2H)-furanone derivative, is extracted from the mushroom *Inonotus sp.* [23]. Recently, inotilone- and rosmanol-reduced CEBPD expression was suggested to attenuate inflammatory responses in murine macrophages upon LPS treatment [22], [23]. To further evaluate whether CEBPD can be a therapeutic target in RA therapy, we tested the effects of inotilone and rosmanol on the migration and proliferation of rFLS and on the tube formation of HUVECs. Either inotilone or rosmanol inhibited the TNFα-induced expressions of CEBPD and its downstream targets (Fig. 7A). Moreover, conditioned medium harvested from the inotilone- or rosmanol-treated THP-1 cells significantly attenuated the TNFα-induced proliferation and migration of rFLS (Fig. 7B and 7C) and HUVEC tube formation (Fig. 7D).

## Discussion

The process of inflammation is virtually identical in various disease states, and CEBPD activation has been observed in many chronic inflammatory diseases. Therefore, CEBPD could be an important mediator of inflammatory disease. The characterization of CEBPD biology in the inflammatory process should open up new avenues for developing novel prognostic markers and strategies in the treatment of inflammatory diseases. However, CEBPD, and especially its downstream targets, remain poorly studied in various inflammation-related diseases. In this study, we demonstrated that CEBPD is indeed involved in RA pathogenesis, at least in part through its downstream targets CCL20, CXCL1, IL23A and TNFAIP6. These CEBPD-responsive secretion factors promoted the migration and proliferation of rFLS, but only CXCL1 and TNFAIP6 contributed to angiogenesis. When we applied the idea of CEBPD inactivation to RA pathogenesis, we observed that the anti-inflammatory molecules and CEBPD inhibitors inotilone and rosmanol exerted inhibitory effects on the migration and proliferation of rFLS and the tube formation of HUVECs.

Although chronic inflammatory cells include activated T and B cells, plasma cells, mast cells and activated macrophages, the central cells of rheumatoid arthritis are macrophages, due to their prominent numbers in the inflamed synovial membrane and at the cartilage-pannus junction. Moreover, the multitude and abundance of macrophage-derived mediators in RA and their paracrine/autocrine effects identify macrophages as local and systemic amplifiers of disease [10]. Herein, to determine the CEBPD-regulated factors in macrophages and characterize their involvement in communication with neighboring cells, we performed a cytokine array and a microarray to identify CEBPD-responsive secretion factors. Two identified cytokines, CCL20 and CXCL1, serve as chemoattractants for leukocytes and certain nonhematopoietic cells [24]–[26]. Our results further demonstrate that both CCL20 and CXCL1 can promote rFLS migration, and importantly, IL-23 and TNFAIP6 have the same effects. IL23 plays a predominant role in autoimmune inflammation [27]. IL-23-deficient mice are resistant to CIA [8], but the precise role of IL-23 in RA pathogenesis remains unclear. IL-23 is a heterodimer that consists of the p40 and p19 (also named IL23A) subunits. The p40 subunit is also a subunit of IL-12. IL-23 and IL-12 may play opposite roles in carcinogenesis. IL-23 promotes tumor immune evasion, but IL-12 has anti-tumor properties [28], [29]. Therefore, CEBPD-induced IL23A could enhance IL-23 signaling over the competing IL-12 signaling, promoting tumorigenesis. However, this speculation remains to be confirmed empirically. In addition, T helper 17 cells (TH17) are a newly discovered subset of T helper cells that produce interleukin 17 (IL-17) and are distinct from TH1 and TH2 cells. TH17 cells are thought to play a key role in autoimmune disease and are significantly increased in RA synovial fluid. IL-17, TGF-β, IL-1β, IL-6 and IL-23 are also important for the polarization of TH17 cells [30]–[33]. These cytokines were elevated in our CEBPD-mediated cytokine array profile. Therefore, it is reasonable to speculate that CEBPD is an upstream mediator of the expression of these important cytokines. Moreover, the inflamed synovium is marked by the proliferation of synoviocytes in the lining layer of the synovium. CCL20 and CXCL1 are involved in tumor growth and TNFAIP6 can regulate the growth of smooth muscle cells [24], [26], [34]. IL-23, however, can promote the proliferation of human memory T cells [32] but has not yet been studied in synoviocytes. These findings may account for the phenomenon of pannus formation during inflammatory conditions.

Inflammation is often associated with angiogenesis, and vascular remodeling is associated with chronic inflammatory disorders [35]. Therefore, recognizing which factors can regulate angiogenesis has become a pivotal issue for the therapy of chronic inflammatory disorders, such as psoriasis, rheumatoid arthritis and inflammatory bowel diseases. In this study, we demonstrate that CEBPD activation in macrophages can promote angiogenesis. Moreover, the CEBPD-responsive factors CXCL1 and TNFAIP6 can explain this important discovery. CXCL1 is involved in microvascular endothelial cell tube formation *in vitro* [36]. However, the observation that TNFAIP6 participates in the regulation of angiogenesis is a novel finding. TNFAIP6, also known as TSG-6, is composed mainly of contiguous LINK and CUB (complement subcomponents C1r/C1s, Uegf, BMP-1) modules and was originally identified as a TNFα-responsive factor. The rapid up-regulation of TNFAIP6 in the presence of the proinflammatory cytokines TNFα and IL-1β is consistent with its involvement in inflammatory processes [34]. The LINK module of TNFAIP6 can interact with the extracellular matrix components glycosaminoglycan and hyaluronan and can have chondroprotective effects in various models of inflammation and arthritis [34], [37]–[39]. The chondroprotective effect of TNFAIP6 in arthritis is mediated through the inhibition of MMPs and aggrecanase enzymes that damage cartilage [38]. Therefore, we speculate that the angiogenic effect of TNFAIP6 may occur through ECM remodeling to achieve regulation of vascular formation. TNFα can induce both pro- and anti-angiogenic factors depending on duration or dose [40]. Briefly, *in vitro* short-term treatment (<4 days) or a high dose of TNFα induces a stronger anti-angiogenic effect, but long-term treatment (>4 days) or a lower dose of TNFα shows a stronger pro-angiogenic effect. In this study, the TNFα treatment showed a marginal effect on the inhibition of angiogenesis (Fig. 4B right panel). However, the attenuation of CEBPD in macrophages indeed decreased the angiogenic effect of TNFα, suggesting that CEBPD contributes more to the pro-angiogenic state in the presence of TNFα signaling. Additionally, comparing the treatments with vs. without TNFα, both CXCL1 and TNFAIP6 contributed to a pro-angiogenic effect, but only CXCL1 was involved in TNFα-mediated pro-angiogenic activity (Fig. 6B, compare sh-CX with and without TNFα). These results suggest that CXCL1 contributes to TNFα-mediated angiogenesis and that TNFAIP6 can function independently in angiogenesis.

CEBPD is expressed at a relatively low level under normal physiological conditions and is up-regulated by a variety of extracellular stimuli [41], [42]. CEBPD has been suggested to play a dual role in controlling the basal level of and induction of gene transcription [21], [43]. Interestingly, the basal levels of mouse *Ccl20* and *Tnfaip6*, but not *Cxcl1* and *Il23a*, transcripts were significantly attenuated in *Cebpd*-deficient primary macrophages. This observation suggests that Cebpd may contribute to the basal regulation of *Ccl20* and *Tnfaip6* transcription and implies that Cebpd plays a functional role in non-inflammatory conditions.

Although CEBPD, similarly to NF-κB and STAT3, is responsive to inflammatory factors, we believe that the functions of CEBPD are not identical to those of NF-κB and STAT3 in inflammatory diseases. This idea compelled us to explore CEBPD function and to apply this knowledge to translational medicine. Rosmanol and inotilone are inflammatory inhibitors that function through the inhibition of *PTGS2* (also named COX-2) and *NOS2* (also named iNOS) gene expression [22], [23]. In this study, we further demonstrated that these chemicals reduce the expression of CEBPD-mediated secretory factors upon TNFα treatment in THP-1 cells. We recently demonstrated that CEBPD-activated PTX3 disables macrophages from removing damaged neurons, which may be a mechanism by which inflammation contributes to the development of AD [44]. As mentioned above, in addition to RA, the induction of CEBPD observed in several age-associated disorders, such as AD [15], atherosclerosis [16] and type 2 diabetes [17]. In this study, CEBPD contributed to RA pathogenesis and its inhibitors rosmanol and inotilone reduced the hallmarks of RA pathogenesis. These results indicate the potential of inotilone and rosmanol as anti-inflammatory drugs and further underscore the importance of CEBPD in RA and other chronic inflammation-related diseases.

## Materials and Methods

### Ethics Statement

All of the studies with live animals were approved by the NCKU Laboratory Animal Center and Use Committee in compliance with the Guide for the Care and Use of Laboratory Animals (NCKULAR, 1987).

### *Cebpd*-deficient Mice, Collagen-induced Arthritis and Assessment of Arthritis

The *Cebpd*-deficient (C57BL/6 background) mice were a kind gift from Dr. Esta Sterneck [45]. All of the mice were used at 10–14 wk of age at the time of experimentation and were age matched. All of the procedures were approved and conducted according to the Institutional Animal Care and Committee Guide of our university animal laboratory center. For immunization, chick type II collagen (Sigma-Aldrich, St. Louis, MO, USA) powder was dissolved to a concentration of 4.0 mg/ml overnight at 4°C in 0.1 M acetic acid. An immunizing emulsion was formed by mixing the type II collagen solution with an equal volume of CFA (Sigma-Aldrich) on ice until the emulsion was thick enough not to disperse on the water surface. Mice were injected i.d. at the base of the tail with a total of 100 µl of emulsion containing 200 µg of CII and 50 µg of M. tuberculosis. No additional boost was given due to little improvement in incidence. All of the mice were examined two to three times per week for the initial visual appearance of arthritis after immunization. Arthritis in each individual limb was graded using the following scoring system: 0, normal; 1, apparent swelling and redness limited to individual digits; 2, swelling in more than one joint; 3, severe redness and swelling of the entire paw, including digits; and 4, a maximally inflamed limb with involvement of multiple joints. The maximum score per mouse was 16. The mice were scored as arthritic if more than one paw had a score >2 [46]. The thickness of the hind paws was measured using a digital gauge caliper. At the end of the experiment, the rear paws and joints were removed, fixed, decalcified, and embedded in paraffin. Joint sections (5 µm) were stained with hematoxylin and eosin (H&E) and examined for histological changes in inflammation, pannus formation, cartilage, and bone damage. Arthritic changes in the ankle were scored as previously described: 0, normal; 1, weak leukocyte infiltration but no erosion; 2, modest infiltration and weak erosion; 3, severe infiltration and invasion of bones; and 4, loss of bone integrity [47].

### Cell Culture and Reagents

The human monocytic leukemia cell lines THP-1 and U937 were cultured in RPMI-1640 containing 10% fetal bovine serum (FBS), 100 µg/ml streptomycin, and 100 units/ml penicillin (complete medium). Both cell lines were gifts from Dr. YS Lin [48], [49]. THP-1 monocytic cells were differentiated into macrophages following growth in medium containing 5 ng/mL PMA for 48 h and then subsequently allowed to differentiate into macrophages. HUVECs were purchased from the Bioresource Collection and Research Center (Taiwan) and were maintained in EGM-2 medium (serum-free, growth factor-free; Clonetics) supplemented with 2% FBS, human fibroblast growth factor-B, human epidermal growth factor, human vascular endothelial cell growth factor, long R insulin-like growth factor-1, ascorbic acid, hydrocortisone and heparin. Synovial tissue was obtained from the knee joints of male Sprague-Dawley rats. The tissue specimens were finely minced and digested by treating with 1 mg/ml collagenase and 0.15 mg/ml hyaluronidase overnight. The cells were then filtered and collected by centrifugation, and the precipitated cells were resuspended in complete medium. After 4 passages, the enriched synovial fibroblasts were used for further experiments as described by Ahn et al. [4]. Mouse bone marrow mononuclear cells were obtained from femurs and tibias and grown in RPMI 1640 medium containing 10% fetal calf serum (FCS) and 25 ng/mL macrophage colony-stimulating factor (M-CSF) (R&D Systems Inc.). For mouse macrophage differentiation, bone marrow mononuclear cells were grown in medium containing M-CSF on the 1^st^ and 3^rd^ days; then, on Day 5, any floating cells were discarded and the adherent bone marrow-derived macrophages were cultured in RPMI 1640 medium with 10% FCS until Day 7 for further experiments. Inotilone and rosmanol were obtained from Dr. MH Pan as described in previous studies [50], [51].

### Conditioned Medium Collection and Inotilone and Rosmanol Treatment

To test the effects of macrophage CEBPD on the migration and proliferation of synoviocytes and on angiogenesis, conditioned medium was collected [52], [53]. Briefly, THP-1 cells were infected with lentivirus containing shCEBPD or shluciferase for 48 h. After the removal of the medium containing uninfected virus, the cells were incubated in complete medium with or without TNFα (20 ng/ml) for 24 h; this medium was then collected as knockdown CEBPD-conditioned medium. The conditioned medium of overexpressed CEBPD was harvested after the THP-1 cells were transfected with a pEGFP-CEBPD expression vector or an empty vector using a MicroPorator MP-100 (Digital Bio, Seoul, Korea) and incubated for 24 h. THP-1 cells were pretreated with 5 nM inotilone or rosmanol for 30 min and then treated with TNFα for 1 h. Next, to avoid contamination and side effects from inotilone or rosmanol, the cells were washed and cultured for 24 h in medium with 10% FBS prior to harvesting the conditioned medium.

### Microarray Analysis

Total RNA was isolated from cells using the TRIzol RNA extraction reagent by Invitrogen (Carlsbad, CA, USA). The samples were subjected to microarray analysis at Phalanx Biotech Group, Inc. (Hsinchu, Taiwan) using an Agilent Human Whole Genome OneArray™ Microarray. Good-quality signals were obtained by filtering for scores with a p-value less than 0.05 in all replicates, an M-value greater than 6 in all signals, and a fold change of more than 1.5. The microarray data were then analyzed by Ingenuity Systems (http://www.ingenuity.com/). For RT-PCR, total RNA was isolated and subjected to reverse transcription with SuperScriptTM III (Cat: #18080-051, Invitrogen). PCR was performed with the pairs of primers listed in Table S2.

### Short Hairpin RNA (shRNA) Assay

The lentiviral expression vectors pLKO.1-shLuciferase, containing 5′-CTTCGAAATGTCCGTTCGGTT-3′; pLKO.1-shCEBPD, containing 5′- GCCGACCTCTTCAACAGCAAT-3′; pLKO.1-shCCL20, containing 5′-TCTTGGATACACAGACCGTAT-3′; pLKO.1-shCXCL1, containing 5′- CGGAAAGCTTGCCTCAATCCT-3′; pLKO.1-shIL23A, containing 5′- ACTCAGGGACAACAGTCAGTT-3′; and pLKO.1-shTNFAIP6, containing 5′- GTGGCGTCTTTACAGATCCAA -3′, were obtained from the National RNAi Core Facility located at the Genomic Research Center of the Institute of Molecular Biology, Academia Sinica. The virus was produced from Phoenix cells using Mirus Bio Trans-IT co-transfected with the pMD2.G and psPAX2 vectors along with the pLKO.1-shRNA expression vectors.

### Reporter Plasmids and Luciferase Assay

The 5′-flanking regions of the *CCL20*, *CXCL1*, *IL23A* and *TNFAIP6* genes were obtained from THP-1 cells using the DNeasy Tissue Kit (QIAGEN, Dusseldorf, Germany). PCR was then performed using the primer pairs shown in Table S2. The PCR products were digested with *Kpn*I and *Hind*III and subcloned into the promoterless pGL3-basic vector. For the reporter assay, THP-1 and U937 cells were transiently transfected with these plasmids using an electroporator (Gene Pulser Xcell total system, Bio-Rad Laboratories, Inc.) according to the manufacturer’s instructions. The total amounts of DNA for each experiment were matched by adding empty vector in all transfection experiments. The luciferase activities of the transfectants were measured by the Luciferase Assay System (Promega Corp., Madison, WI) according to the manufacturer’s instructions after 24 h of incubation. For the knockdown approach using oligonucleotides, the siCEBPD oligo, 5′- CCGGGCTGTCGGCTGAGAACGAGAACTCGAGTTCTCGTTCTCAGCCGACAGCTTTTT -3′, and the siLacZ oligo, 5′- CCGGGCGCTAATCACGACGCGCTGTCTCGAGACAGCGCGTCGTGATTAGCGCTTTTTG -3′, were used.

### ChIP Assay

The ChIP assay was conducted essentially as described by Wang *et al.* [43]. Briefly, THP-1 cells were treated with 1% formaldehyde for 15 min. The cross-linked chromatin was then prepared and sonicated to an average size of 500 bp. The DNA fragments were immunoprecipitated with antibodies specific for CEBPD or control rabbit immunoglobulin G (IgG) at 4°C overnight. After reversal of the cross-linking, the immunoprecipitated chromatin was amplified using the primers listed in Table S2, targeting specific regions of the *CCL20*, *CXCL1*, *IL23A* and *TNFAIP6* genomic loci. The amplified DNA products were resolved by agarose gel electrophoresis and were confirmed by sequencing.

### Human Protein Cytokine Array

The Biotin Label-based Human Antibody Array I glass chip (RayBiotech, Norcross, GA) was blocked with a blocking buffer. Using Biotin for labeling, the conditioned medium harvested from CEBPD-overexpressed THP-1 cells was prepared and mixed with the internal control. After removing the blocking buffer, the biotin-labeled sample was added and then incubated at room temperature for 2 h. Following three aspiration/wash steps, each array was incubated with streptavidin-conjugated fluorescence for 2 h. Finally, the slides were aspirated and washed, and the chambers were discarded. The dried slides were scanned and imaged using the Cy3 channel of a GenePix microarray scanner.

### *In vitro* Cell Migration and Cell Proliferation Assays

The QCM™ 96-well Haptotaxis Cell Migration Assays (Chemicon, USA) were used for migration analysis according to the manufacturer’s instructions. Briefly, FLS (1×10^6^ cells/well) were placed in the upper surface of the membrane with serum-free RPMI medium, and the conditioned medium was placed in the lower well. After 24 h incubation, the non-invading FLS were removed from the upper surface of the membrane by gentle swabbing. The cells in the lower surface of the membrane were harvested and analyzed according to the manufacturer’s instructions. The FLS and HUVEC proliferation were determined using a Cell Counting Kit-8 (CCK-8), a sensitive colorimetric assay for viable cells, according to the manufacturer’s protocol (Dojindo Laboratories, Kumamoto, Japan).

### *In vitro* Endothelial Tube Formation Assay

The tube formation assay was performed as follows: 24-well plates were pre-coated with Matrigel and incubated at 37°C to promote gelling. HUVECs were resuspended in EBM-2 medium (serum-free) and conditioned medium was added to each well. The growth medium was used as a positive control. After 24 h incubation, the plates were fixed with 4% paraformaldehyde and a blinded observer assessed the morphology of the tubes. Each condition and experiment was repeated at least three times. Tube-like structures were quantified by counting the number of intersections between branches of the endothelial cell networks in the whole field. The values are represented as the means ± SE of the intersection numbers of three independent experiments.

### Histochemistry and Immunohistochemical Staining

The rear paws and joints were fixed, decalcified and embedded in paraffin. Then, 8-µm sections were stained with H&E for histopathological examination. For immunohistochemical staining, paraffin sections were deparaffinized, blocked with 3% normal goat serum and incubated with antibodies against Ki-67 (M306; springbio.com), CD31 (M3380; springbio.com), and F4/80 (BM8; Santa Cruz, CA, USA) at 37°C for 1 hour. Subsequently, a peroxidase DAB detection system (PK6200; Vector Laboratories) was applied according to the manufacturer’s instructions. The sections were counterstained with hematoxylin. The data were quantified as described by Chien *et al.* [54]. Briefly, ten high-power fields were examined per section. Scores of 1 to 3 were assigned to samples with no membrane staining but with cytoplasmic staining, intermediate membrane staining, and strong membrane staining, respectively. Samples without positive staining were assigned a score of 0. All of the sections were independently scored by two pathologists. The sections were observed under a fluorescence microscope.

### Western Blotting

Cells were lysed in modified RIPA buffer (50 mM Tris-HCl (pH 7.4), 150 mM NaCl, 1 mM EDTA, 1% NP40, 0.25% sodium deoxycholate, 1 mM dithiothreitol (DTT), 1 µg/ml aprotinin and 1 µg/ml leupeptin). Following lysis, the lysates were resolved on a sodium dodecyl sulfate (SDS)-containing 10% polyacrylamide gel, transferred to a polyvinylidene difluoride (PVDF) nylon membrane, and probed with specific antibodies at 4°C overnight. Antibodies against CEBPD (M-17) and β-actin were purchased from Santa Cruz Biotechnology (Santa Cruz, CA, USA) and Sigma (St. Louis, MO, USA), respectively. Specific bands were detected by a horseradish peroxidase-conjugated antibody and revealed by an enhanced chemiluminescence (ECL) Western blot system (Pierce, Rockford, IL, USA).

### Statistical Analysis

The data are expressed as the means ± SD of at least three independent experiments. The differences in the histopathological scores and clinical scores between groups were analyzed using the Mann-Whitney U test for parametric variables due to the relatively small number of experimental mice. A value of *p*<0.05 was considered to indicate statistical significance.

## Supporting Information

Figure S1 **CEBPD downstream targets mediated the effect of proliferation on HUVEC.** We used overexpression and knockdown CEBPD strategy to harvested supernatants as conditioned medium to measure CEBPD downstream targets effects on HUVEC. HUVEC were treated with conditioned medium that have noted above for 24 hours and used CCK-8 to measure cells viability. Data were represented as the mean ±SE of three independent experiments performed in triplicate. Asterisks represent statistical differences (*P<0.05; Student’s t test).
(TIF)

Figure S2 **The effect and regulators among CEBPD, CCL20, CXCL1, IL23A and TNFAIP6.** A, CEBPD stimulates CCL20, CXCL1, IL23A and TNFAIP6 promoters activity. The u937 cells were transiently transfected with CCL20 (-1000 to +50), CXCL1 (−970 to +51), IL23A (−1200 to +60) or TNFAIP6 (−600 to +76) luciferase reporter gene and co-transfected with pcDNA3/HA/CEBPD or pcDNA3/HA empty plasmids for 24 hours. The luciferase assay was performed as described in Materials and methods. Data are represented as the mean ±SE of relative luciferase activity from the three independent experiments in duplicate. B, Knockdown efficiency of lentiviral shCCL20(Sh-CC), shCXCL1(Sh-CX), shIL23A(Sh-IL) and shTNFAIP6(Sh-TA) were examined by RT-PCR assay. THP-1 cells were infected with lentiviruses as indicated for 24 h. Later, after treatment with TNFα, the experimental cells were divided to harvest total RNA for RT-PCR analysis and to collect conditioned media for the migration, proliferation and tube formation assays.
(TIF)

Table S1 **The candidates of CEBPD-regulated genes: comparing CEBPD-regulated profile.** The global profiling was performed by Agilent human whole genome oligo 4X 44 K array. Among over four hundred genes, fold>1.5; p<0.05 was considered significant, responded to lentiviruses bearing shCEBPD or shLuciferase and TNFα-treated THP-1 cells.
(TIF)

Table S2 **Primers used for RT-PCR, PCR cloning of promoter and chromatin immunoprecipitation.**
(TIF)
