# Supplementary figures and images for: Role of Macrophage CCAAT/Enhancer Binding Protein Delta in the Pathogenesis of Rheumatoid Arthritis in Collagen-Induced Arthritic Mice
Source: PLoS One. 2012 Sep 24;7(9):e45378. doi: 10.1371/journal.pone.0045378 (PMC3454428; doi:10.1371/journal.pone.0045378)

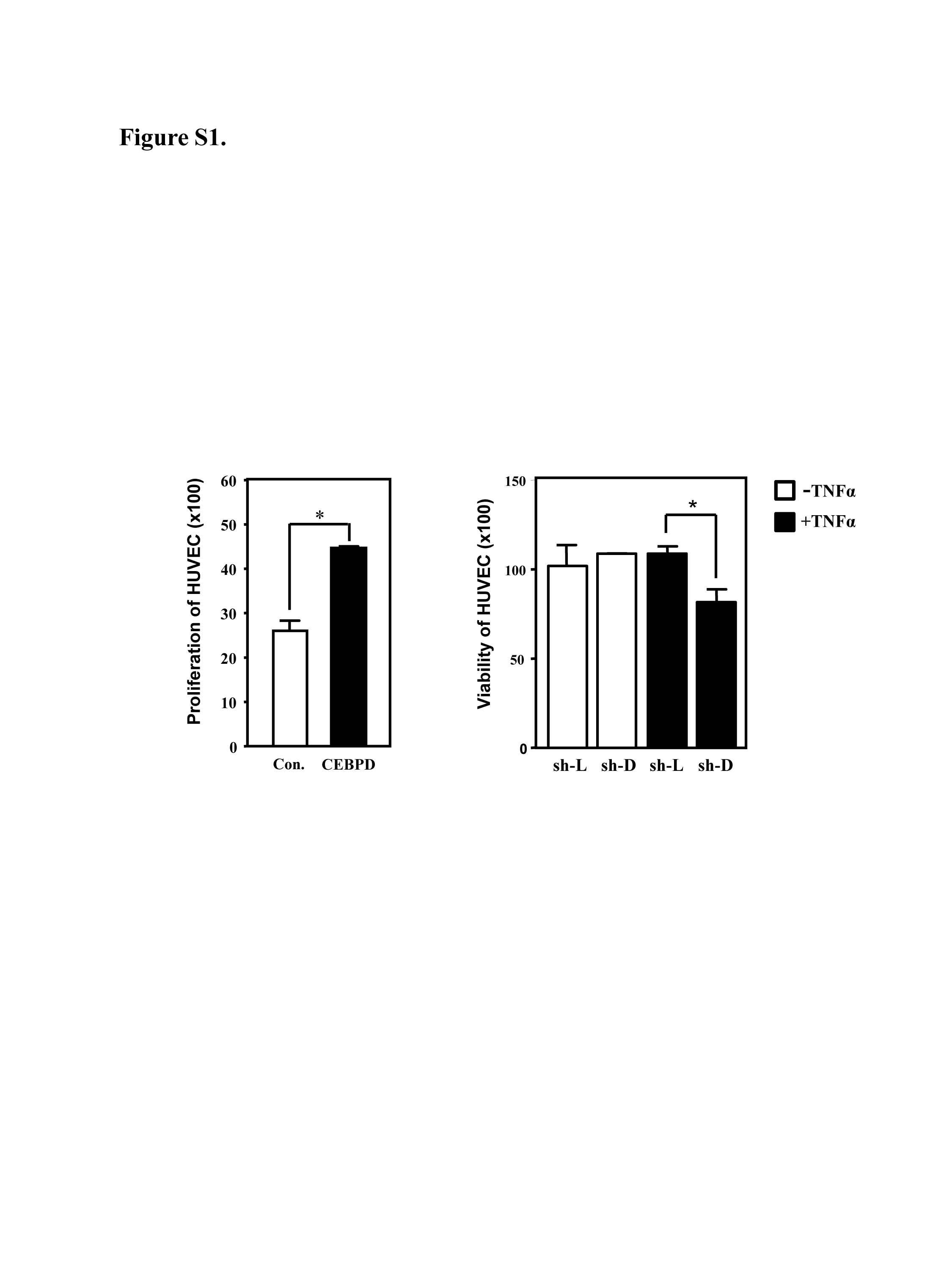

Supplement: Figure S1 — CEBPD downstream targets mediated the effect of proliferation on HUVEC. We used overexpression and knockdown CEBPD strategy to harvested supernatants as conditioned medium to measure CEBPD downstream targets effects on HUVEC. HUVEC were treated with conditioned medium that have noted above for 24 hours and used CCK-8 to measure cells viability. Data were represented as the mean ±SE of three independent experiments performed in triplicate. Asterisks represent statistical differences (*P<0.05; Student’s t test). (TIF) [file pone.0045378.s001.tif]

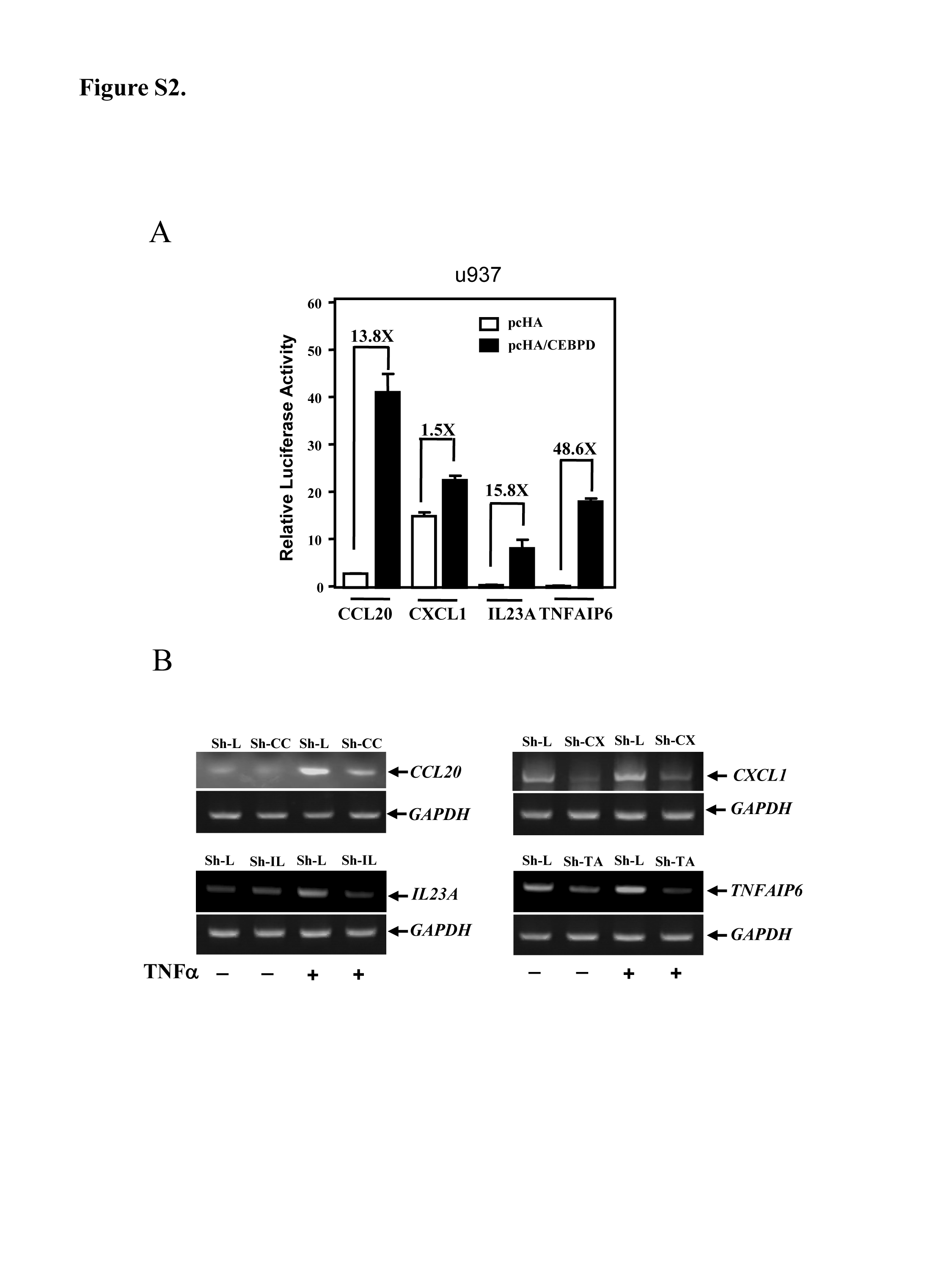

Supplement: Figure S2 — The effect and regulators among CEBPD, CCL20, CXCL1, IL23A and TNFAIP6. A, CEBPD stimulates CCL20, CXCL1, IL23A and TNFAIP6 promoters activity. The u937 cells were transiently transfected with CCL20 (-1000 to +50), CXCL1 (−970 to +51), IL23A (−1200 to +60) or TNFAIP6 (−600 to +76) luciferase reporter gene and co-transfected with pcDNA3/HA/CEBPD or pcDNA3/HA empty plasmids for 24 hours. The luciferase assay was performed as described in Materials and methods. Data are represented as the mean ±SE of relative luciferase activity from the three independent experiments in duplicate. B, Knockdown efficiency of lentiviral shCCL20(Sh-CC), shCXCL1(Sh-CX), shIL23A(Sh-IL) and shTNFAIP6(Sh-TA) were examined by RT-PCR assay. THP-1 cells were infected with lentiviruses as indicated for 24 h. Later, after treatment with TNFα, the experimental cells were divided to harvest total RNA for RT-PCR analysis and to collect conditioned media for the migration, proliferation and tube formation assays. (TIF) [file pone.0045378.s002.tif]

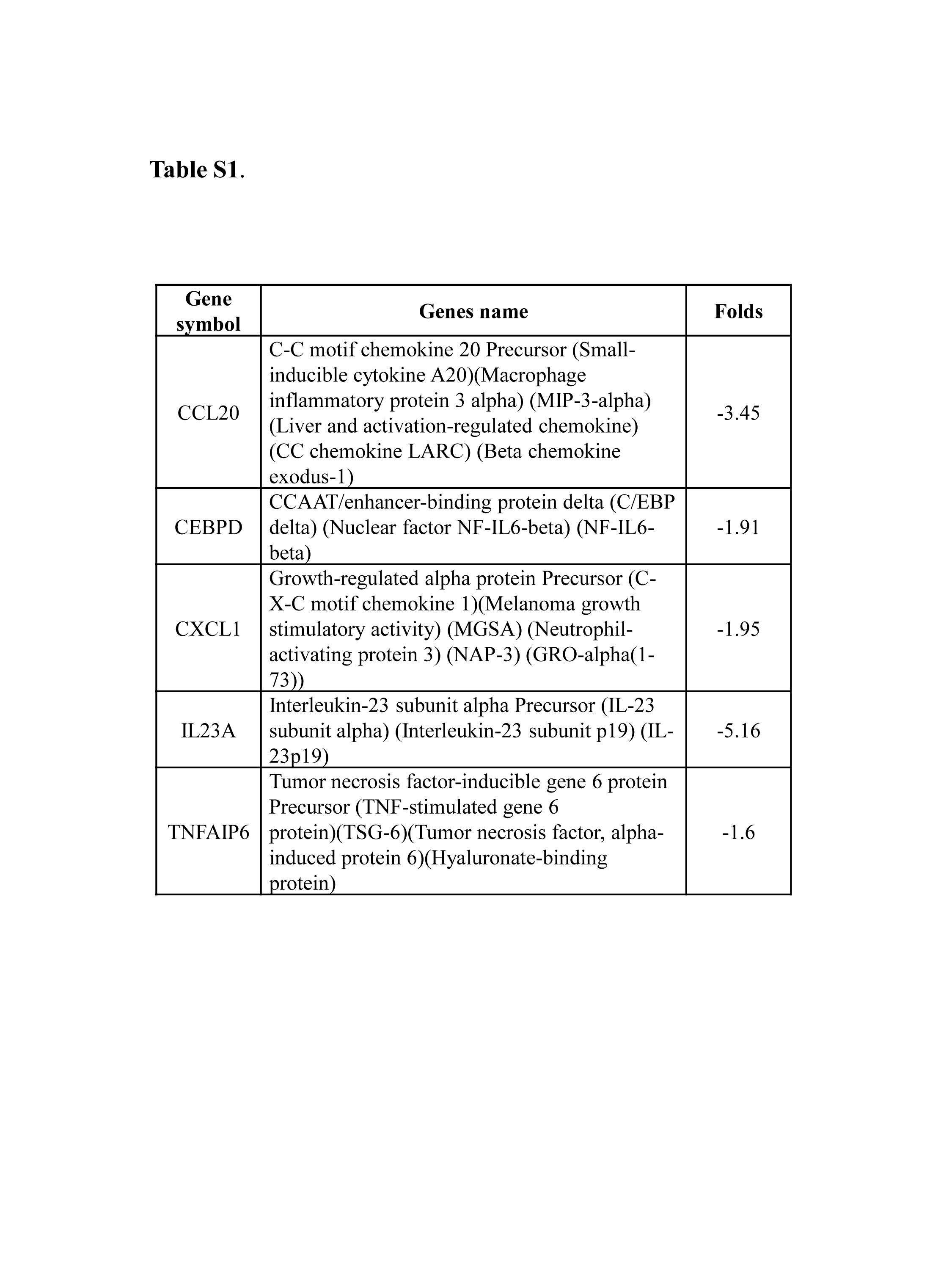

Supplement: Table S1 — The candidates of CEBPD-regulated genes: comparing CEBPD-regulated profile. The global profiling was performed by Agilent human whole genome oligo 4X 44 K array. Among over four hundred genes, fold>1.5; p<0.05 was considered significant, responded to lentiviruses bearing shCEBPD or shLuciferase and TNFα-treated THP-1 cells. (TIF) [file pone.0045378.s003.tif]

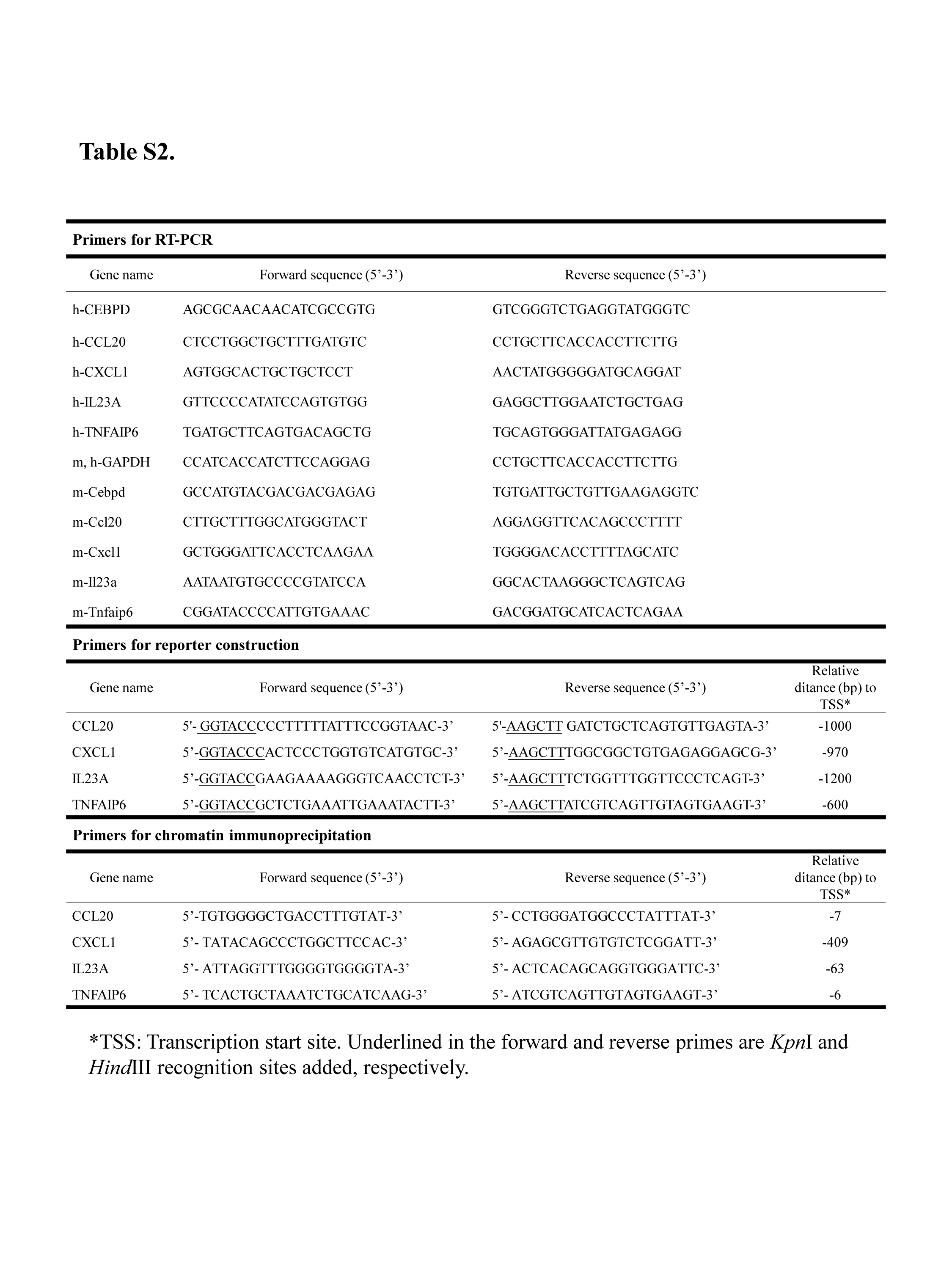

Supplement: Table S2 — Primers used for RT-PCR, PCR cloning of promoter and chromatin immunoprecipitation. (TIF) [file pone.0045378.s004.tif]
